# Illness uncertainty, psychological well-being and psychological resilience in people with schizophrenia

**DOI:** 10.1192/j.eurpsy.2025.326

**Published:** 2025-08-26

**Authors:** G. Sahin-Bayindir, S. Gunay, S. Er, B. K. Gultekin

**Affiliations:** 1 Florence Nightingale Faculty of Nursing, Department of Mental Health and Psychiatric Nursing, Istanbul University - Cerrahpasa; 2 Psychiatric Clinic; 3 Pul Clinic, Erenkoy Mental Health and Neurology Training and Research Hospital, Istanbul, Türkiye

## Abstract

**Introduction:**

Patients with schizophrenia experience uncertainty due to unpredictable symptoms, disease progression and lack of information about the illness, treatment and prognosis. Studies underline that illness uncertainty is a risk factor in schizophrenia, while psychological well-being and quality of life are critical outcomes. Psychological resilience is also recognised as having a mediating role.

**Objectives:**

The aim of this study was to examine the relationships between illness uncertainty, psychological resilience and psychological well-being in people with schizophrenia.

**Methods:**

In this cross-sectional study, 150 participants with a diagnosis of schizophrenia (DSM-5) were recruited. Data were collected using the Mishel Uncertainty in Illness Scale-Community Form, the Psychological Well-being Scale and the Connor-Davidson Resilience Scale. Data were analysed using descriptive statistics, independent samples t-test, one-way ANOVA and pearson correlation analysis.

**Results:**

The mean age of people diagnosed with schizophrenia who participated in the study was 37.52±10.62 years, 41.3% (n=62) were high school graduates, 68.7% (n=103) were single, 53.3% (n=80) had income equal to expenses, 59.3% (n=89) were not working, 54.7% (n=82) lived with their parents and 38.7% (n=58) had children. The study found a negative and weak relationship between the mean MUIS-C total score and the mean PWBS total score (r=-0.359), and also a negative and weak relationship between the mean MUIS-C total score and the mean CD-RISC total score (r=-0.287).

**Conclusions:**

It is important for health professionals to assess the uncertainty experienced by people with schizophrenia, to examine its impact on their psychological well-being and to understand how they cope with their illness.

**Disclosure of Interest:**

None Declared

